# Human papillomavirus and other genital infections in indigenous women from Paraguay: a cross-sectional analytical study

**DOI:** 10.1186/1471-2334-13-531

**Published:** 2013-11-09

**Authors:** Laura Mendoza, Pamela Mongelos, Malvina Paez, Amalia Castro, Isabel Rodriguez-Riveros, Graciela Gimenez, Patricia Araujo, Gloria Echagüe, Valentina Diaz, Florentina Laspina, Wilberto Castro, Rosa Jimenez, Ramón Marecos, Santiago Ever, Gerardo Deluca, María Alejandra Picconi

**Affiliations:** 1 Department of Public Health and Epidemiology, Health Sciences Research Institute (IICS), National University of Asuncion (UNA), Rio de la Plata y Lagerenza, 1120, 2511, Asunción, Paraguay; 2 Department of Clinical and Microbiological Analysis, IICS, UNA, Asunción, Paraguay; 3 Cervical Pathology Department. Faculty of Medical Sciences, UNA, Asunción, Paraguay; 4 Department of Immunology, IICS, UNA, Asunción, Paraguay; 5 Health Center of Pozo Colorado, Ministry of Public Health, Department of Presidente Hayes, Asunción, Paraguay; 6 Regional Hospital of Villa Hayes, Ministry of Public Health, Department of Presidente Hayes, Asunción, Paraguay; 7 Molecular Applications Laboratory, Faculty of Medicine, Northeast National University, Corrientes, Argentina; 8 Oncogenic Viruses Service, National Institute of Infectious Diseases (INEI) - ANLIS ”Dr. Malbrán”, Buenos Aires, Argentina

**Keywords:** Human papillomavirus, Indigenous Paraguayan women, Genital infections, Cervical inflammation

## Abstract

**Background:**

The incidence of cervical cancer in Paraguay is among the highest in the world, with the human papillomavirus (HPV) being a necessary factor for cervical cancer. Knowledge about HPV infection among indigenous women is limited. This cross-sectional study analyzed the frequency of HPV and other genital infections in indigenous Paraguayan women of the Department of Presidente Hayes.

**Methods:**

This study included 181 sexually active women without cervical lesions. They belonged to the following ethnicities: Maká (n = 40); Nivaclé (n = 23); Sanapaná (n = 33); Enxet Sur (n = 51) and Toba-Qom (n = 34). The detection of HPV and other gynecological infectious microorganisms was performed by either molecular methods (for *Mycoplasma hominis, Ureaplasma urealyticum, Chlamydia trachomatis*), gram staining and/or culture (for *Gardnerella vaginalis, Candida sp, Trichomonas vaginalis, Neisseria gonorrhoeae*), serological methods (for *Treponema pallidum*, human immunodeficiency virus [HIV]) or cytology (cervical inflammation).

**Results:**

A high prevalence (41.4%) of women positive for at least one sexually transmitted infection (STI) was found (23.2% any-type HPV, 11.6% *T pallidum*, 10.5% *T vaginalis*, 9.9% *C trachomatis* and 0.6% HIV) with 12.2% having more than one STI. HPV infection was the most frequent, with 16.1% of women positive for high-risk HPV types. There was a statistically significant association observed between any-type HPV and *C trachomatis* (p = 0.004), which indicates that the detection of one of these agents should suggest the presence of the other. There was no association between any-type HPV and other genital infections or cervical inflammation, suggesting that other mechanism could exist to favor infection with the virus.

**Conclusion:**

This multidisciplinary work suggests that STIs are frequent, making it necessary to implement control measures and improve diagnosis in order to increase the number of cases detected, especially in populations with poor access to health centers.

## Background

Cervical cancer is second globally as a malignant tumor among women and accounts for 9.8% of all cancer cases. According to 2008 data, Paraguay ranks seventh in the incidence of cervical cancer in Latin America, with an incidence rate of 35.0/100,000 women-years and a mortality rate of 16.6/100,000 women-years [1].

Human papillomavirus (HPV) is the causative agent of cervical intra-epithelial neoplasia (CIN) and cervical cancer and is the most common sexually transmitted viral infection worldwide [2]. There are more than 100 types of HPV, including 40 types that exclusively infect the cervical mucosa. Based on the known epidemiological associations with cervical cancer, HPV is categorized as high-risk HPV (HR-HPV), as low-risk HPV (LR-HPV) or in other unclassified groups [3].

However, there is evidence on the role of other sexually transmitted agents as co-factors for the development of cervical cancer in HPV-positive women. *Chlamydia trachomatis* and human immunodeficiency virus (HIV) are the two most studied of these agents [4-7]. It was suggested that the increased risk of cervical cancer in women co-infected with *C trachomatis* is due, in part, to an inflammatory response associated with free radical generation and the development of genetic instability [6]. Other organisms such as *Gardnerella* sp, *Candida* sp, *Trichomonas* sp, *Mycoplasma hominis*, *Ureaplasma urealyticum* and *Treponema pallidum* have also been associated with cervical inflammatory processes, a situation that may facilitate the entrance of HPV [8,9].

Molecular methods, such as polymerase chain reaction (PCR), have permitted the detection of infectious agents from the genital tract that are difficult or impossible to isolate by conventional techniques, allowing for a gain in sensitivity and specificity. This is true for HPV as well as other infectious agents such as *Ureaplasma parvum*, *U urealyticum*, *C trachomatis*, *M hominis* and *Mycoplasma genitalium*[10-12].

According to data from the Directorate General of Surveys, Statistics and Census of 2008, ethnic populations of Paraguay have a fertility rate of 6.3 children per woman and a low education level, with 38.9% of the population over 14 years of age being illiterate. Furthermore, most indigenous communities have difficulty in accessing health care. All these factors, in conjunction with chronic HPV infection, could favor the development of cervical cancer [13,14].

The Department of Presidente Hayes in Paraguay has the largest indigenous population, representing 23% of the total population with 108,600 inhabitants, based on data from the Directorate General of Surveys, Statistics and Census of 2008 [13]. Only a few studies on the detection of sexually transmitted infections (STIs) (e.g., HIV, syphilis and hepatitis B virus) have been conducted in Paraguayan indigenous women [15,16].

The aim of this study was to determine for the first time the frequency of HPV infection and other genital infections among indigenous women from Paraguay.

## Methods

This analytical cross-sectional study included 181 indigenous women, sexually active, not pregnant and with no medical or surgical treatment during the study period, belonging to the Department of Presidente Hayes, Paraguay.

While there are national centers for primary health care offering cytology (Papanicolaou [Pap] smear) at no cost, most indigenous women cannot access these centers. In this context and in order to get closer to these women for the opportunity to determine their status regarding STIs, the working group contacted the authorities of the Regional Hospital of Villa Hayes, which is located near the areas inhabited by indigenous communities included in this study. They coordinated three medical visits to each indigenous community. The visits were organized as follows: the first was to contact the Cacique and indigenous community leaders to explain the objectives of the study and obtain approval for the study; the second was to inform women of the objectives of the study, the study conditions and requirements, the potential benefits to be gained and to invite them to participate. Finally, the third visit was made to obtain cervical samples from those women who had agreed to participate.

Visits were made to women who belonged to the Qemkuket community of ethnic Maká; the Novoctas community of ethnic Nivacle; the Laguna Pato Complex of ethnic Sanapaná; the Maxhawaya and Espinillo communities of ethnic Enxet South; and finally the Rio Verde and Toba-Qom communities belonging to ethnic Toba-Qom.

The study protocol (P11/2010) was approved by the Ethics Committee of the Institute for Research in Health Sciences of the National University of Asunción. All women signed an informed consent written in Spanish prior to the sampling of biological material and the application of a questionnaire to collect data related to socio-demographic and sexual characteristics of indigenous women. In the case of indigenous women who did not speak Spanish, informed consent was translated to her language by a community member. Furthermore, in the case of women under 18 years old, informed consent was signed by the parent or guardian. The results of the study were presented at the local hospital (Regional Hospital of Villa Hayes), whose area of influence are the indigenous localities included in this study, for monitoring and treatment. All women with treatable infections received appropriate treatment. In particular, in the case of HPV studies, the following criteria were adopted for clinical management: women who tested positive for HPV but had negative cytology result would be followed with a cytology control in a year; and women who tested positive for both HPV and cytology would be referred for colposcopy.

The study material collected included:

A blood sample for serology (syphilis screening and detection of HIV).

A sample of vaginal secretions for *Gardnerella vaginalis*, *Candida* sp and *Trichomonas vaginalis* analysis.

Two endocervical brushes, one for the molecular detection of HPV, *M hominis*, *C trachomatis* and *U urealyticum* and the other for cervical cytology screening.

A cervical swab for detection of *Neisseria gonorrhoeae* by isolation in Thayer-Martin medium.

Collection of blood samples, vaginal secretions and endocervical brushes was made by medical doctors or nurses at the Health Post of the indigenous communities or in cases where there were no Health Posts, at the home of the family. Any biological material collected was appropriately transported and stored until processing.

### Cytology (Papanicolaou [Pap] smear)

Cervical brushing was performed by a trained gynecologist and placed in a slide correctly identified, which was referred to the Health Sciences Research Institute (IICS), National University of Asunción (UNA) for analysis. The interpretation of the findings and categorization of results were reported according to the Bethesda System 2001 [17].

### HPV genotyping by PCR-Reverse Line Blot hybridization (RLB)

Cervical cells were collected using a conical brush, which was placed into a tube with a preservative solution (DNA collection device Hybrid Capture 2, Digene Corporation, Gaithersburg, USA). The sample was subsequently stored at −70°C until processing.

The DNA extraction from cervical samples was performed by the method described by Mendoza et al. [18]. The quality of the extracted DNA was verified by PCR amplification of a 268 bp fragment of the gene for β-globin gene using the primers PC04 and GH20 [19].

The viral genome detection was performed by a generic PCR using consensus primers PGMY 09/11, which amplify a 450 bp fragment of L1 viral gene [20]. HPV genotyping was performed by the RLB (CHUV) method, as previously described by Estrade et al. [20], using the type-specific oligoprobes corresponding to 37 HPV types (HPVs 16, 18, 31, 33, 35, 39, 45, 51, 52, 56, 58, 59, 66, 68, 6, 11, 26, 34, 40, 42, 43, 44, 53, 54, 55, 57, 61, 70, 71 [CP8061], 72, 73, 81 [CP8304], 82/MM4, 82/IS39, 83 [MM7], 84 [MM8] and CP6108). The classification of HPV as high-risk types was performed according to Muñoz et al. [3]; HPV 16, 18, 31, 33, 35, 39, 45, 51, 52, 56, 58, 59, 68, 73 and 82 were included in this group. In addition, HPV 26, 53 and 66 were included as probable HR-HPV.

### Detection of other genital infections

Vaginal secretions (*G vaginalis, Candida* sp. and *T vaginalis)* and endocervical swab samples *(*for *N gonorrhoeae)* were taken, placed in Stuart transport medium and sent to the IICS Microbiology Laboratory, where a smear, Gram stain and culture on appropriate media were performed [21].

Syphilis was detected from a blood sample using the serological test Venereal Disease Research Laboratory (VDRL test, Wiener lab, Rosario, Argentina). All VDRL-positive samples were confirmed by fluorescent *T pallidum* antibodies (FTA-ABS, slide Trepospot BioMerieux, Marcy l'Etoile, Francia; conjugated IgG BioMeriex, Marcy l'Etoile, Francia).

HIV was detected using a fourth generation enzyme immunoassay (ELISA), used as per the manufacturer’s instructions, for determination of antibodies to HIV types 1 and 2 and the p24 antigen of HIV-1 in human serum and plasma (DIA.PRO Diagnostic Bioprobes SRL, Milán, Italy). HIV-positive samples were submitted for a second ELISA and subsequently confirmed by RecomLine HIV-1 and HIV-2 IgG (Mikrogen Diagnostik, Neuried, Germany).

*M hominis, C trachomatis, U urealyticum* were detected from DNA extracted from genital samples and were analyzed by multiplex-PCR according to the protocol described by Golshani et al. [11].

### Statistical analysis

The sample size calculation to determine the frequency of HPV was made using an estimated prevalence of 30% with a 95% confidence interval, a width of 7% and a total population of indigenous women of the Presidente Hayes Department over 10 years of age of 9,492 [22]. The required sample size was 162 women. Considering the Directorate General of Surveys, Statistics and Census, which showed that the different ethnic groups in this study had common features, such as a high fertility rate (6.3 children per woman) and a low education level (a mean of 2.2 years of school), we decided to make a single sample size calculation including women of different ethnicities [13,22].

The analysis was performed using descriptive and analytical statistics. The association between proportions was assessed by chi-square analysis using Epi Info™ 7.1.1.14 (Centers for Disease Control and Prevention, Atlanta, USA). For all data analyses conducted, p values < 0.05 were considered statistically significant.

## Results

**Figure 1:**
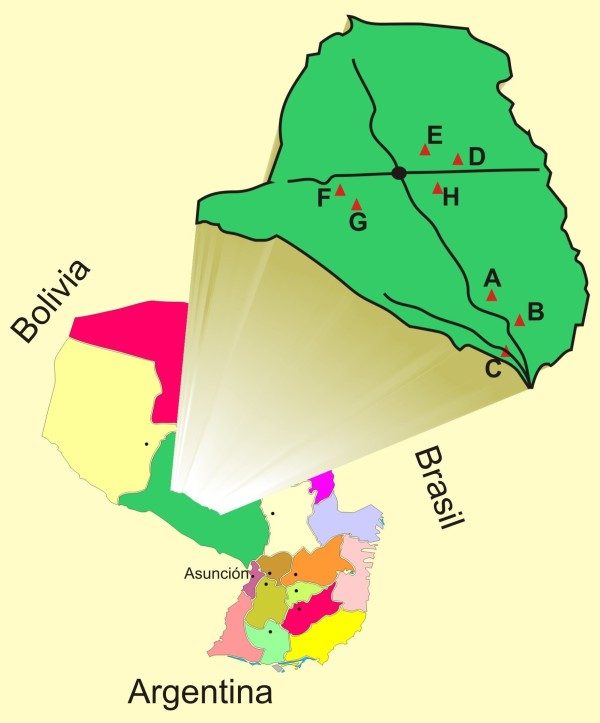
**Map of Presidente Hayes Department, Paraguay.** Participating communities are marked by triangles; **A.** Rio Verde (52 km from Asuncion); **B.** Tobaqom (51 km from Asuncion); **C.** Quemkuket (30 km from Asuncion); **D.** Maxhawaya (326 km from Asuncion); **E.** Laguna Pato Complex (320 Km from Asuncion); **FyG.** Novoctas (434 km from Asuncion) and **H.** Espinillo (297 km from Asuncion).

Of the 181 women enrolled, 40 women belonged to the Qemkuket community of ethnic Maká; 23 to the Novoctas community (15 Marcelo Cue, 8 Duarte Cue) of ethnic Nivacle; 33 to the Laguna Pato Complex (9 Lolaico”i, 13 Lolaico guasú, 3 Brillante, 7 Laguna Pato, 1 Salado) of ethnic Sanapaná; 28 to Maxhawaya (5 Monte Alto, 23 Maxhawaya) and 23 to Espinillo (13 Espinillo, 10 Pozo Colorado), both communities of ethnic Enxet South; and 16 women of Rio Verde and 18 women of Toba-Qom belonging to ethnic Toba-Qom. Figure 1 shows the map of Presidente Hayes Department, including the study participants’ communities. The communities visited ranged between 30 and 434 km in distance from Asunción, the capital of Paraguay.

The demographic characteristics, as well as the reproductive and sexual history of the 181 women included in this study, are shown in Table 1. The median age was 30 years (interquartile range, 23–41), with 47% (85/181 women) being younger than 30. The observed illiteracy rate was 39%, with a median of 2 years of school. A high percentage of women (60%) used hormonal contraceptives. Notably, 71.8% of indigenous women participants underwent a Pap smear for the first time.

**Table 1:** Socio-demographic and sexual characteristics of indigenous women

**Characteristics**	**Total (n = 181 women)**
**Age (years)**	
Median (Interquartile range)	30 (23–41)
**Education (years)**	
Median (Interquartile range)	2 (0–4)
Illiterate n (%)	73 (40.3)
Elementary school n (%)	95 (52.5)
High school n (%)	13 (7.2)
**Age at 1st sexual intercourse (years)**	
Median (Interquartile range)	16 (13–19)
≤16 n (%)	105 (58.0)
> 16 n (%)	76 (42.0)
**n sexual partners**	
Median (Interquartile range)	1 (1–2)
1 n (%)	107 (59.1)
2-3 n (%)	62 (34.2)
≥4 n (%)	12 (6.6)
**Pregnancies**	
Yes n (%)	165 (91.1)
No n (%)	16 (8.8)
**n pregnancies**	
Median (Interquartile range)	3 (2–5)
1-3 n (%)	85 (51.1)
4-6 n (%)	49 (29.7)
>6 n (%)	31 (17.1)
**Oral contraceptive use**	
Yes n (%)	107 (59.1)
No n (%)	74 (40.9)
**Smoking**	
Yes n (%)	39 (21.5)
No n (%)	142 (78.5)
**Previous Cytology**	
Yes n (%)	51 (28.2)
No n (%)	130 (71.8)

A high frequency of STIs was detected, 41.4% (95% CI 34.2%-49%), primarily as HPV (any type), syphilis, *C trachomatis*, *T vaginalis* and HIV. In 12.2% (95% CI 7.8%-17.8%) of the women studied, more than one STI was detected, although any-type HPV infection was the most frequent. Table 2 shows the frequency of genital infections.

**Table 2:** **Frequency of genital infections detected in indigenous women of the Department of Presidente Hayes, Paragua**y

	**n (%)**	**95% CI**
**n Total**	**181 (100)**	
HPV	42 (23.2)	17.3-30.0
HR-HPV^*^	29 (16.1)	11.1-22.3
Probable HR-HPV**	7 (3.9)	1.6-7.8
Syphilis (VDRL & IgG***)	21 (11.6)	7.3-17.2
*Trichomonas vaginalis*	19 (10.5)	6.4-15.9
*Chlamydia trachomatis*	18 (9.9)	6.0-15.3
HIV	1 (0.6)	0-3.0
STI total****	75 (41.4)	34.2-49
**More than 1 STIs**	22 (12.2)	7.8-17.8
**Other genital infection**		
*Gardnerella vaginalis*	83 (45.9)	38.4-53.4
*Mycoplasma hominis*	56 (30.9)	24.3-38.2
*Ureaplasma urealyticum*	37 (20.4)	14.8-27.1
*Cándida sp*	13 (7.2)	3.9-12.0

CI: Confidence Interval; HPV: human papillomavirus; HR-HPV: high-risk oncogenic HPV; HIV: human immunodeficiency virus; STI: sexually transmitted infection (HPV, VDRL, *Trichomonas vaginalis, Chlamydia trachomatis,* HIV); VDRL: Venereal Disease Research Laboratory.

*Frequency of positive samples for at least one type of HR-HPV.

**Frequency of positive samples for at least one type of probable HR-HPV.

***Confirmed by Immunoglobulin G (IgG).

**** Frequency of women with at least one sexually transmitted infection.

The prevalence of any-type HPV DNA was 23.2% (95% CI 17.3%-30%) and was 16.1% (95% CI 11.1%-22.3%) among HR-HPV positive women. A higher frequency of HR-HPV was detected in women in the age ranges of 13 to 29 years (18.8%) and over 49 years (23.1%). Table 3 shows the frequency of HR-HPV in relation to age.

**Table 3:** Frequency of HR-HPV by age of indigenous women of the Department of Presidente Hayes, Paraguay

	**Age (years)**			
	**<30**	**30-39**	**40-49**	**≥50**
**n Total**	**85**	**45**	**25**	**26**
**Frequency of HR-HPV n (%)**	16 (18.8)	5 (11.1)	2 (8.0)	6 (23.1)

HR-HPV: high-risk oncogenic human papillomavirus.

According to cytological diagnosis, all 181 women were negative for cervical lesions; however, 13.8% (95% CI 9.1%-19.7%) of women presented with cervical inflammation. There was no significant association observed between the presence of inflammation and any-type HPV infection.

In addition, there was a statistically significant association between the concomitant presence of any-type HPV and *C trachomatis* infection (p = 0.004). No significant association was found between HPV and *M hominis*, *U urealyticum*, *T vaginalis*, *G vaginalis* or *Candida* sp infection. Table 4 shows the distribution of genital infections and cervical inflammation according to HPV results.

**Table 4:** Frequency of genital infections and cervical inflammation according to any human papillomavirus results of indigenous women

**Variables**	**Frequency of genital infections**				**P-value**
	**HPV positive women (n = 42)**		**HPV negative women (n = 139)**		
	**n (%)**	**95% CI**	**n (%)**	**95% IC**	
*Gardnerella vaginalis*	20 (47.6)	32.0-63.6	63 (45.3)	36.9-54.0	p = 0.974
*Mycoplasma hominis*	16 (38.1)	23.6-54.4	40 (28.8)	21.4-37.1	p = 0.254
*Ureaplasma urealyticum*	11 (26.2)	13.9-42.0	26 (18.7)	12.6-16.2	p = 0.292
Syphilis (VDRL & IgG*)	3 (7.1)	1.5-19.5	18 (12.9)	7.9-19.7	p = 0.304
*Trichomonas vaginalis*	5 (11.9)	4.0-25.6	14 (10.1)	5.6-16.3	p = 0.734
*Chlamydia trachomatis*	9 (21.4)	10.3-36.8	9 (6.5)	3.0-11.9	**p = 0.004**
*Cándida sp*	2 (4.8)	0.6-16.2	11 (7.9)	4.0-13.7	p = 0.489
HIV	-		1 (0.7)	0-3.9	-
Cervical inflammation	5 (11.9)	4.0-25.6	20 (14.4)	9.0-21.3	P = 0.683

HPV: human papillomavirus, CI: Confidence Interval, VDRL: Venereal Disease Research Laboratory.*Confirmed by IgG.

## Discussion

There are a few epidemiological studies of HPV detection and probable co-factors associated with the acquisition of viral infection in indigenous populations in South America [23-27]. This is the first study for the detection of HPV, *C trachomatis* and other sexually transmitted agents in the indigenous population of Paraguay, and it may serve as a precedent for future epidemiological investigations of STIs.

Any-type HPV and HR-HPV were detected in 23.2% and 16.1% of the total women studied, respectively. These results are comparable to those found by Mendoza et al. [18]. That series found a 20.8% prevalence of any-type HPV and 13.5% for HR-HPV in Caucasian women with negative cytology. However, these results are lower than the frequency detected in studies performed with indigenous women from Misiones (64% any-type HPV and 59% HR-HPV) and Jujuy (52% any-type HPV), Argentina and indigenous from the Brazilian Amazon (42.85% any-type HPV) and Venezuelan Amazon (57% any-type HPV) [23-25,27].

This disparity may be due to dissimilarity between the demographic characteristics and sexual behaviors, among others, of the participants, which could differentiate the risk of acquisition of the viral infection in these populations. The indigenous women analyzed in this study had an average age of 30 years and an average of 3 pregnancies, and most of them (59%) declared only one sexual partner. In contrast, in the population analyzed by Tonon et al. [23], the indigenous women participants had a median age of 15 years, and 61% of them were less than 30 years. It is well known that HPV infection is age dependent, being most common in women younger than 25 years old [28]. This could partly explain the lower prevalence of HPV observed in the present work. In addition, populations of indigenous women from Argentina and Brazil presented with other features, such as a high frequency of polygamy and of pregnancies among other co-factors. Although monogamy by women is not enough for limiting the risk of STIs, because the partner’s behavior is also crucial, polygamy would favor the acquisition of an STI [23-25,27].

However, the prevalence of any-type HPV detected in this study (23.2%) was higher than the 13.2% (95% CI 12.7%-13.7%) observed in a meta-analysis including 17,500 urban women from South America with normal cytology [28]. This difference could be due to geographical parameters, ethnicity, sexual behavior and the techniques used for the HPV detection, among other factors. It should be taken into account that the detection of HPV infection in young women (primarily <30 years old) is mostly at the expense of transient infections that clear rapidly [29]; the relatively increased HPV prevalence detected in the indigenous women with a median age of 30 years could have more chance of being associated with persistent infections, which might represent a risk factor for cervical cancer development in this population.

Another important finding in this study was that all 181 women were negative for cervical lesions. These data are consistent with those provided by the Central Laboratory of Cytodiagnosis of the Ministry of Public Health for 2001 and 2006, which detected a frequency of abnormal Pap tests of 0.5% and 1.1%, respectively, in urban Paraguayan women [30]. However, it should be noted that cytology is a subjective and poorly reproducible test with limited sensitivity that requires regular repetition to achieve the desired efficacy [31]. Furthermore, Sankaranarayanan et al. [32] observed a significantly low sensitivity of cytology in women aged 25 to 39 years (p < 0.001); this range of age includes most of the women in the present study. In this context, it cannot be ruled out that the some cervical abnormalities could have been missed.

A high frequency of STIs (41.4%) was detected, with 12.2% presenting with more than one sexually transmitted organism. Despite the fact that most indigenous women (59.1%) declared only one sexual partner, their male partners are probably polygamous, which represents a higher risk of acquiring an STI, in an unfavorable scenario of difficult access for infections treatment.

The frequency of STIs detected were higher than that observed in the study by Oliveira et al. [9], which, in northern Brazil, detected a frequency of 19.6% (95% CI 16.5%-23.2%) in Caucasian immunocompetent women with at least one STI (HPV, *C trachomatis*, *N gonorrhoeae*, *T vaginalis* or syphilis). These present results are also lower than those reported by Menendez et al. [33], who detected a frequency of 70% (95% CI 64%-76%) in women from rural areas of Mozambique, Africa with at least one STI, including HIV-positive women. Although in the present study cervical lesions were not detected, the results suggest that STIs are frequent in indigenous women and, therefore, these infections should be checked periodically as they are considered risk factors for the development of cervical cancer and other pathologies.

The significant association found in this study between any-type HPV and *C trachomatis* is in agreement with Smith et al. [34], who observed that among HPV DNA-positive cases and controls, the risk of invasive cervical cancer was almost twice as high in *C trachomatis* seropositive than in seronegative women (odds ratio [OR] = 1.8; 95% CI 1.2-2.7), after adjustment for age, center, oral contraceptive use, history of Pap smears, number of full-term pregnancies and herpes simplex virus 2 seropositivity. In addition, da Silva Barros et al. [7] suggested that *C trachomatis* seropositivity seemed to be associated with the severity of cervical neoplasia in women infected with HPV, principally when HPV types 16 and 18 were involved. Therefore, it may be important to screen women infected with HPV for co-infection with *C trachomatis*, bearing in mind that this association may have synergistic pathological effects.

It is suggested that the increased risk of developing cervical cancer in women co-infected with microorganisms mentioned above is the result of an inflammatory response [6]; but the mechanism by which these co-infections favor the development of cervical cancer is not yet clear [35]. In the present study, an association between cervical inflammation and HPV was not detected, which suggests that other mechanism may be involved.

It was notable that no women positive for *N gonorrhoeae* were identified. This may be due to the methodology of the culture used, which has some disadvantages especially in settings where long distance travel makes maintenance of conditions for the optimal performance of the culture difficult [36]. These results suggest that testing and diagnosis of *N gonorrhoeae* must be improved in order to enhance the number of cases detected, especially in populations with a high frequency of other STIs and with poor access to health centers.

## Conclusions

Finally, despite not having detected cervical lesions in the indigenous women included in this study, a high frequency of HPV and other STIs was observed. In addition, this work suggests that in HPV-positive women, the presence of *C trachomatis* should be evaluated. These data confirm that screening for genital infections may be important to reveal the simultaneous presence of different STIs and facilitate the adoption of measures for treatment, monitoring and disease prevention. At the same time, this multicenter study helped to implement new molecular techniques for detection of these infections in the Paraguayan laboratory (IICS-UNA), which will be available for use in other health centers.

## Competing interests

The authors declare that they have no competing interests.

## Authors’ contributions

LM conceived the study and wrote the manuscript. PM, GD and MAP performed the molecular detection of HPV, *C trachomatis*, *M hominis* and *U urealyticum* and analyzed the results. AM, GE, VD, FL collected the samples (cervical brush, blood, vaginal secretions) and analyzed the results, and GE, VD, RJ performed the serological test and analyzed the results. MIR, GG, IRR, PA, MP performed the surveys, obtained informed consent and analyzed the data collected. WC performed the cytology study and interpreted the results. SE, RM participated in organized visits to indigenous communities, in the recruitment of indigenous women and in the delivery of results and treatment when necessary. All authors participated to the design of the study and were involved in drafting and revising the manuscript. All authors read and approved the final manuscript.

## Pre-publication history

The pre-publication history for this paper can be accessed here:

http://www.biomedcentral.com/1471-2334/13/531/prepub
